# Neuronal Inflammation is Associated with Changes in Epidermal Innervation in High Fat Fed Mice

**DOI:** 10.3389/fphys.2022.891550

**Published:** 2022-08-23

**Authors:** David S. Umbaugh, J. Claire Maciejewski, Joshua S. Wooten, Brianne L. Guilford

**Affiliations:** Department of Applied Health, Southern Illinois University Edwardsville, Edwardsville, IL, United States

**Keywords:** peripheral neuropathy, prediabetes, intraepidermal nerve fiber density, von Frey, mechanical allodynia, TrkA, insulin resisitance

## Abstract

Peripheral neuropathy (PN), a debilitating complication of diabetes, is associated with obesity and the metabolic syndrome in nondiabetic individuals. Evidence indicates that a high fat diet can induce signs of diabetic peripheral PN in mice but the pathogenesis of high fat diet-induced PN remains unknown.

**PURPOSE**: Determine if neuronal inflammation is associated with the development of mechanical hypersensitivity and nerve fiber changes in high fat fed mice.

**METHODS:** Male C57Bl/6 mice were randomized to a standard (Std, 15% kcal from fat) or high fat diet (HF, 54% kcal from fat) for 2, 4, or 8 weeks (*n* = 11-12 per group). Lumbar dorsal root ganglia were harvested and inflammatory mediators (IL-1α, IL-1β, IL-2, IL-3, IL-4, IL-5, IL-6, IL-10, IL-12p70, IL-17, MCP-1, IFN-γ, TNF-α, MIP-1α, GMCSF, RANTES) were quantified. Hindpaw mechanical sensitivity was assessed using the von Frey test. Intraepidermal nerve fiber density (IENFD) and TrkA nerve fiber density were quantified via immunohistochemistry.

**RESULTS:** After 8 weeks, HF had greater body mass (33.3 ± 1.0 vs 26.7 ± 0.5 g, *p* < 0.001), fasting blood glucose (160.3 ± 9.4 vs 138.5 ± 3.4 mg/dl, *p* < 0.05) and insulin (3.58 ± 0.46 vs 0.82 ± 0.14 ng/ml, *p* < 0.001) compared to Std. IL-1α, RANTES and IL-5 were higher in HF compared to Std after 2 and 4 weeks, respectively (IL-1α: 4.8 ± 1.3 vs 2.9 ± 0.6 pg/mg, *p* < 0.05; RANTES: 19.6 ± 2.2 vs 13.3 ± 1.2 pg/mg *p* < 0.05; IL-5: 5.8 ± 0.7 vs 3.1 ± 0.5 pg/mg, *p* < 0.05). IENFD and TrkA fiber density were also higher in HF vs Std after 4 weeks (IENFD: 39.4 ± 1.2 vs 32.2 ± 1.3 fibers/mm, *p* < 0.001; TrkA: 30.4 ± 1.8 vs 22.4 ± 1.3 fibers/mm). There were no significant differences in hindpaw sensitivity for Std vs HF.

**CONCLUSION:** Increased inflammatory mediators preceded and accompanied an increase in cutaneous pain sensing nerve fibers in high fat fed mice but was not accompanied by significant mechanical allodynia. Diets high in fat may increase neuronal inflammation and lead to increased nociceptive nerve fiber density.

## Introduction

The International Diabetes Foundation estimates that 537 million adults suffer from diabetes and this number is projected to reach 783 million by 2045 (Foundation, 2021). The global prevalence of prediabetes is even greater and is estimated at 100 million cases in the United States alone (Centers for Disease Control and Prevention, 2020). Neuropathy, a syndrome characterized by damage to the autonomic or peripheral nervous system, is the most common complication of diabetes (Feldman et al., 2019), affecting up to 50% of diabetic individuals (Callaghan et al., 2015). Notably, prediabetic patients also develop peripheral neuropathy (Lu et al., 2013; Ziegler et al., 2014) and patients with prediabetes and the metabolic syndrome are at increased risk for developing neuropathy (Kazamel et al., 2021).

Distal symmetric polyneuropathy is the most common type of neuropathy (Feldman et al., 2019) and will be referred to as peripheral neuropathy (PN) throughout this publication. PN manifests in a stocking and glove type pattern, affecting the hands and feet (Feldman et al., 2019). Typically, nerve fibers are affected in length-dependent manner; the longer, distal axons that innervate the feet are first affected (Tracy and Dyck, 2008; Stino and Smith, 2017) causing increased sensitivity to touch or loss of sensation (Boulton et al., 2005). PN can affect small-diameter thinly myelinated or unmyelinated fibers and/or large myelinated fibers.

A proportion of patients suffer from small-fiber PN, in which pain sensing A-delta and C fibers are impacted. Symptoms of small fiber PN include increased thermal sensitivity and pain sensitivity which may present as pins and/or needles, burning, aching, or stinging sensations (Stino and Smith, 2017). Reduced or abnormal intra-epidermal nerve fiber density (IENFD) is a widely accepted diagnostic measure of small fiber PN (Divisova et al., 2012; Stino and Smith, 2017). Interestingly, small fiber PN is the most common form of PN in patients with prediabetes and early stage type 2 diabetes (Divisova et al., 2012) and is associated with the metabolic syndrome (Kazamel et al., 2021).

High fat fed nondiabetic mice also exhibit symptoms of small fiber PN, characterized by increased mechanical sensitivity (mechanical allodynia) and changes in IENFD (Groover et al., 2013; Xie et al., 2013; Obrosov et al., 2017). Although streptozotocin-induced type 1 diabetic (STZ) mice exhibit reduced IENFD, (Christianson et al., 2003a; Kennedy and Zochodne, 2005), high fat fed nondiabetic mice with mechanical hypersensitivity exhibit no change in IENFD (Obrosova et al., 2007; Guilford et al., 2011; Xu et al., 2014), or increased peptidergic pain-sensing tropomyosin receptor kinase A receptor expressing (TrkA) nerve fibers despite no change in IENFD (Groover et al., 2013; Zhang et al., 2018). Thus, this shift towards the TrkA population of nociceptive nerve fibers may be responsible for the onset of mechanical hypersensitivity in rodents and humans. Despite this novel finding, most clinical and experimental studies assess IENFD but do not assess TrkA nerve fiber density. The current study aims to fill this gap by assessing both IENFD and TrkA nerve fiber density at multiple points in the time course of development of mechanical hypersensitivity in high fat fed nondiabetic mice.

It was initially purported that diabetic PN develops due to a combination of consequences resulting from chronic hyperglycemia including oxidative stress (Chung et al., 2003; Feldman, 2003), mitochondrial dysfunction (Brownlee, 2001; Vincent et al., 2010), and microvascular ischemia (Chung and Chung, 2005; Oltman et al., 2005). However, recent evidence suggests that factors independent of hyperglycemia (i.e. dyslipidemia, inflammation, obesity, etc.) may contribute to the development of PN, in prediabetic and diabetic patients. The high fat diet-induced prediabetic obese rodent model has become useful for evaluating factors that may contribute to the development of PN in nondiabetic and prediabetic individuals.

Although growing evidence indicates both nondiabetic and insulin resistant rodents and humans PN (Papanas et al., 2011; Stino and Smith, 2017), the mechanisms underlying the pathogenesis of PN without excess hyperglycemia are poorly understood.

Low-grade inflammation is associated with the pathogenesis of diabetes, occurs in prediabetes (Duksal et al., 2016), and has been proposed as a mechanism contributing to PN progression (Zeng et al., 2018; Zhang et al., 2018). Additionally, it is well established that a high fat diet in nondiabetic humans and rodents is associated with elevated pro-inflammatory mediators in the serum (Erridge et al., 2007; Calder et al., 2011; Herieka and Erridge, 2014). Thus, inflammation may be the linking factor between high fat feeding and the development of PN in rodents. Similarly, inflammation is a plausible connection between increased risk of PN in obesity, the metabolic syndrome, and prediabetes. Although the aforementioned studies have established a relationship between inflammation and PN, only one study has assessed inflammation the peripheral nervous system in a high fat fed nondiabetic model of PN (Zhang et al., 2018).

The primary aim of the current study was to assess indices of PN (mechanical hypersensitivity and nerve fiber density) and pro- and anti-inflammatory mediators in the lumbar dorsal root ganglia (nerve cell bodies of the peripheral nerves that innervate the hindpaws) at several timepoints to determine if neuronal inflammation precedes or occurs at the onset of PN symptoms. We hypothesized that elevated pro-inflammatory mediators would precede increased nociceptive TrkA nerve fiber density and mechanical hypersensitivity. Positive results would point to inflammation is a potential initiating mechanism behind the onset of mechanical hypersensitivity and nerve fiber changes associated with high fat diet-induced PN.

## Materials and methods

### Animals and diet

Seven-week-old male C57BL/6 mice were purchased from Charles River (Wilmington, MA) acquired and housed two per cage, kept under a 12:12 h light/dark cycle, and given *ad libitum* access to food and water. All mice consumed a standard diet before arriving at the Exercise Physiology laboratory at Southern Illinois University Edwardsville. After baseline testing, mice were randomly assigned to a standard diet (14% kcal from fat) or a high fat diet (54% kcals from fat) and further randomized to the 2-weeks, 4-weeks, or 8-weeks intervention (*n* = 11–12 mice per diet per each time point). At the end of the 2-weeks, 4-weeks, and 8-weeks dietary interventions, mice were deeply anesthetized by isofluorane inhalation and euthanized by decapitation. All procedures were approved by the Southern Illinois University Edwardsville Animal Care and Use Committee and were compliant with the National Research Council (US) Committee for the Update of the Guide for the Care and Use of Laboratory Animals.

### Body mass, glucose, and insulin

Body mass and fasting glucose and insulin levels were obtained at baseline and end study for each dietary intervention (2-weeks, 4-weeks, or 8-weeks). Three hours prior to blood collection, food was removed from the cage and blood was collected via tail snip. Blood glucose was measured using glucose diagnostic reagents (Sigma, St. Louis, MO). Blood samples for insulin were clotted on ice for 30 min, centrifuged at 7,000 x *g*, and serum was removed and stored at −80°C until analysis. Serum samples were thawed and insulin levels were quantified using ELISA following the manufacturer’s instructions (Mercodia, Winston Salem, NC).

### Mechanical sensitivity

Mechanical sensitivity was assessed using von Frey behavioral testing. Mice were placed on an elevated (55 cm above table) wire mesh platform and individually enclosed in a transparent, plastic cage. The 1.4 g von Frey monofilament was applied 6 times to each hindpaw footpad. The testing was done in a pattern such that the filament was applied to one foot of every mouse, mice were undisturbed for 3 minutes, then application of the filament to the opposite foot of the first mouse in the rotation began. A positive response was recorded only when the mouse withdrew the hindpaw in response to the stimulus. Each mouse underwent a total of 12 filament applications and percent withdrawal was calculated by taking the number of positive responses divided by the total number (12) of applications. Percent withdrawal was used to calculate group means.

### Inflammation

The lumbar dorsal root ganglia (DRG) house the nerve cell bodies that innervate the hindpaws. Lumbar DRG were harvested following euthanization at end study for each intervention (2-weeks, 4-weeks, and 8-weeks). DRG were sonicated in 75 μl of lysis buffer (137 mM NaCl (58.44 g/mol), 20 mM Triaminomethane (121.14 g/mol), 1% NP-40, 10% glycerol) that included protease inhibitors [1 mM PMSF, 1 μg/ml leupeptin (Research Products International, Prospect, Illinois), 0.5 mM sodium otrthovandate, 10 μg/ml aprotinin (Sigma Aldrich, St. Louis, Missouri) and proteins were extracted for 1 h by vortexing at 10-min intervals. The sample was then centrifuged at 7,000 x *g* for 10 min. Frozen tissue homogenates were shipped on dry ice to Quansys Biosciences (Logan, UT) for analysis via custom multiplex ELISA. The multiplex ELISA plate was coated with 16 capture antibodies specific to the pro and anti-inflammatory mediators: IL-1α, IL-1β, IL-2, IL-3, IL-4, IL-5, IL-6, IL-10, IL-12p70, IL-17, MCP-1, IFN-γ, TNF-α, MIP-1a, GMCSF, and RANTES.

### Intraepidermal nerve fiber density

Hindpaw footpad skin was dissected following euthanization after the 2, 4, and 8-weeks dietary interventions and post-fixed for 2 h in Zamboni’s Fixative (Newcomer Supply, Inc., Cat # 1,459, Middleton, WI) rinsed overnight in 1% PBS (pH 7.4 at 4°C), and immersed for 24 h in 30% sucrose in 1X PBS (pH 7.4 at 4°C). The hindpaw footpads were then serial sectioned (30 μm) using a Leica CM 1860 cryostat, mounted on SuperFrost Plus slides (Fisher Scientific, Pittsburg, PA), and stored at −20°C.

Fluorescence immunohistochemistry was used to assess total epidermal innervation and TrkA specific nerve fibers. Total nerve fibers were labelled with rabbit anti-human PGP 9.5 primary antibody (Bio-Rad, Hercules, CA; 1:80,000) and peptidergic nociceptive nerve fibers were labeled with goat anti-rat TrkA (R + D Systems, Minneapolis, Minnesota; 1:250). The Tyramide Signal Amplification (TSA) kit (Perkin Elmer, Boston, MA) was utilized to enhance the PGP 9.5 signal and reduce background. The TSA kit requires use of a biotin secondary antibody (Jackson Immuno Research) and the streptavidin-HRP (included in the kit) followed by a tyramide conjugate to amplifiy the PGP 9.5 signal. The specific procedure is described below.

After a 5-min thawing period, the slide mounted tissue was coated in 3% H_2_O_2_ for 10 min, washed 1 × 5 min in PBS, then covered with preincubation solution (1.5% normal donkey serum, and 0.5% triton X-100 in Superblock [Thermo Fisher, Waltham, MA]) for 1 h at room temperature. Slides then were incubated overnight in both primary antibodies (anti-PGP 9.5 and anti-TrkA) diluted in Superblock at 4°C. Slides were washed the following day (3 × 5 min in PBST) followed by 1-h incubation at room temperture in secondary antibodies (biotin-SP long spacer affinpure donkey anti-rabbit IgG [Jackson Immuno Research, West Grove, PA, 1:1,600]; and ALexa Fluor donkey anti-goat 488 [ThermoFisher, United States, 1:2000]) diluted in preincubation solution and SuperBlock. Slides were then washed (3 × 5 min in PBST) and then coated in streptavadin-HRP (included in TSA kit, Perkin Elmer, Boston, MA) diluted in Superblock. After 30-min incubation at room temperature, the slides were washed (2 × 5 min in PBST) followed by 1 × 5 min in PBS. Cyanine three tyramide conjugate (Perkin Elmer, Boston, MA) was diluted (1:50) in amplification buffer (Perkin Elmer, Boston, MA) and pipetted to cover the slides for a 10-min incubation period. Slides were washed (2 × 5 min in PBST) followed by a 1 x 5-min wash in PBS. Slides were cover slipped with PBS and stored in a humidity chamber at 4°C for ≤24 h before images were captured.

Fluorescent digital images of the epidermal/dermal border were acquired using a Nikon Eclipse CI-E microscope and Nikon Elements software. Three fields of view from each section and three sections per slide were used to manually count nerve fibers that crossed the epidermal-dermal border. Eighteen Z-stack images at 20x magnification were captured per mouse (9 red, nine green). Z-stack images were viewed in extended depth of focus. The nerve fibers that cross the outermost layer of skin (epidermis) and the inner layer of skin (dermis) were counted separately in each red image (PGP 9.5, total nerve fibers) and each green (TrkA specific fibers) image.

### Statistical analyses

Two factor analysis of variance (ANOVA) with Fisher’s test of least square difference *post hoc* comparisons were used to assess differences within each group at the different time points (2-week vs 4-weeks, vs 8-weeks). Statistical significance was set at *p* < 0.05.

## Results

### Body mass, glucose, and insulin

**FIGURE 1:**
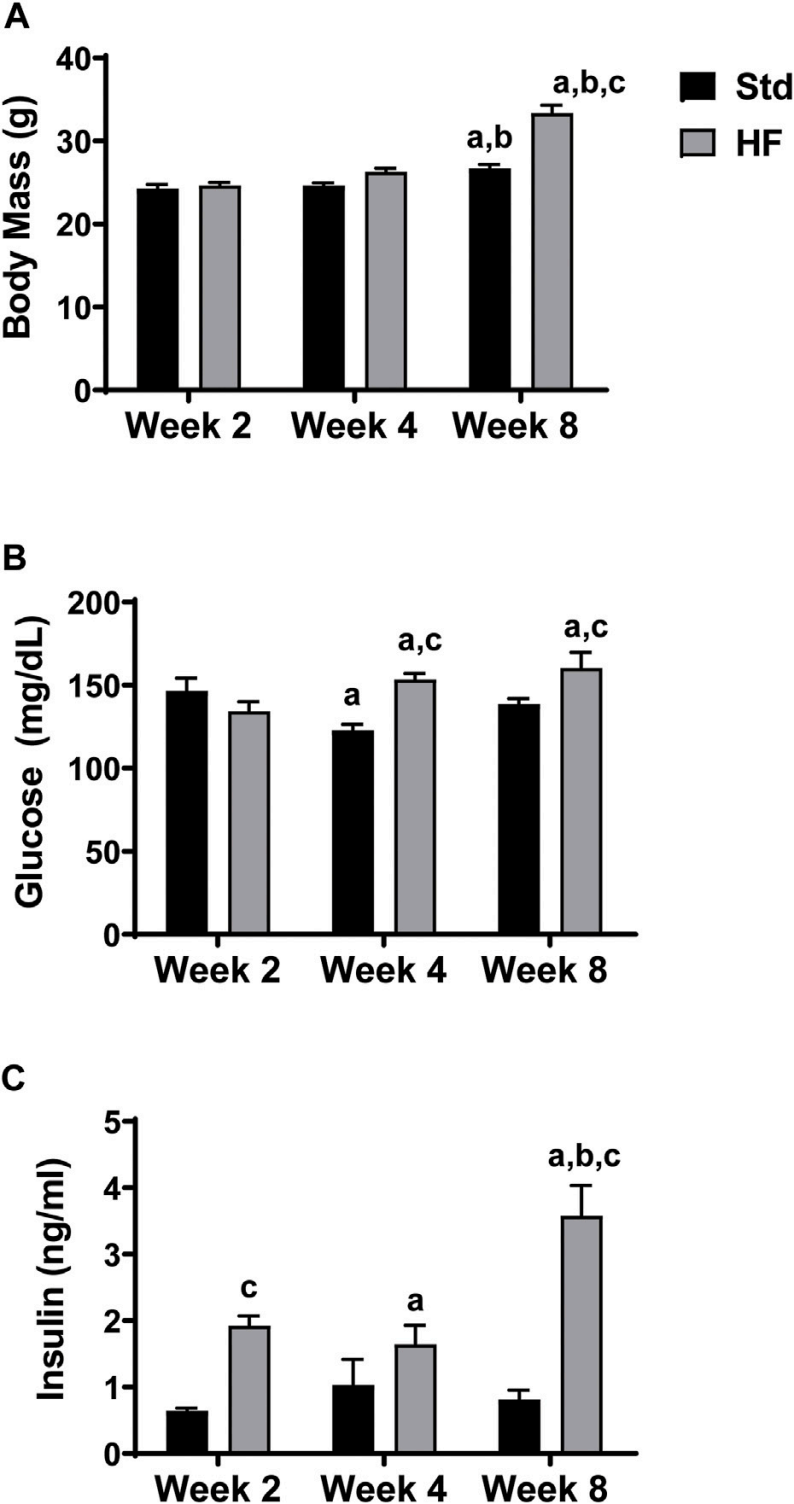
Body Mass and Fasting Blood Glucose and Insulin Levels. HF exhibit greater body mass **(A)**, fasting blood glucose **(B)**, and insulin **(C)** levels compared to Std after 8 weeks of dietary intervention. ^a^ *p* < 0.05 vs wk 2 within diet, ^b^ *p <* 0.05 vs wk 4 within diet, and ^c^ *p* < 0.05 for Std vs HF within the week. Data are presented as mean ± SEM (*n* = 11–12 mice per group).

Both Std (26.7 ± 0.5 g) and HF (33.3 ± 1.0 g) had greater body mass at week 8 compared to their same diet counterparts at week 2 (Std: 24.2 ± 0.5, *p* = 0.004; HF: 24.6 ± 0.4 g, *p* = 0.000) and week 4 (Std: 24.6 ± 0.3, *p* = 0.014; HF: 26.3 ± 0.4 g, *p* = 0.000). Body was also greater in HF compared to Std (*p* = 0.000) at week 8 (Figure 1A), but there were no significant differences in body mass at week 2 or week 4 in Std vs HF. Following the same pattern as body mass, HF had greater blood glucose (Std: 138.5 ± 3.3, HF: 160.3 ± 9.4 mg/dl, *p* = 0.014) and insulin (Std: 0.8 ± 0.1, HF: 3.5 ± 0.5 ng/ml, *p* = 0.000) levels at 8 weeks compared to Std (Figures 1B,C). HF fed mice had higher insulin levels after just 2 weeks of HF feeding (Std: 0.6 ± 0.0, HF: 1.9 ± 0.1 ng/ml, *p* = 0.003) whereas the diet did not significantly impact glucose levels until week 4 (Std: 122.7 ± 3.8, HF: 153.4 ± 3.7 mg/dl, *p* = 0.001).

### Mechanical sensitivity

**FIGURE 2:**
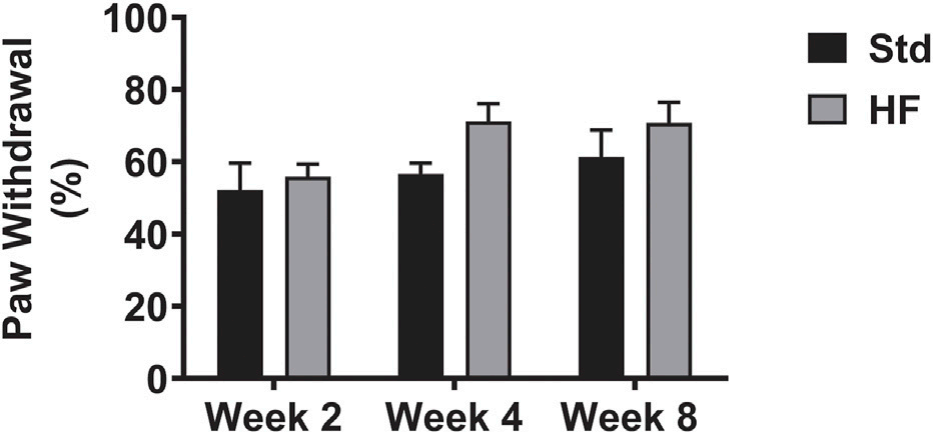
Hindpaw Mechanical Sensitivity. Data are presented as mean ± SEM (*n* = 11–12 mice per group). No significant differences.

There were no significant differences between diet groups at any timepoint or within diet groups between timepoints (Figure 2). Notably, paw withdrawal did peak at the 4-weeks timepoint in the HF group (Week 2: 55.8 ± 3.5%, Week 4: 71.2 ± 4.8%, Week 8: 70.8 ± 5.7%) and the *p* value was 0.066 for week 2 vs week 4 in the HF group, thus trending towards reaching statistical significance. In addition, diet group differences were greatest (although did not reach statistical significance) at week 4 (Std: 56.7 ± 3.0, HF: 71.2 ± 4.8%, *p* = 0.082) compared to week 2 (Std: 52.2 ± 7.4, HF: 55.8 ± 3.5%, *p* = 0.666) and week 8 (Std: 61.4 ± 7.5, HF: 70.8 ± 5.7%, *p* = 0.232).

### Nerve fiber density

**FIGURE 3:**
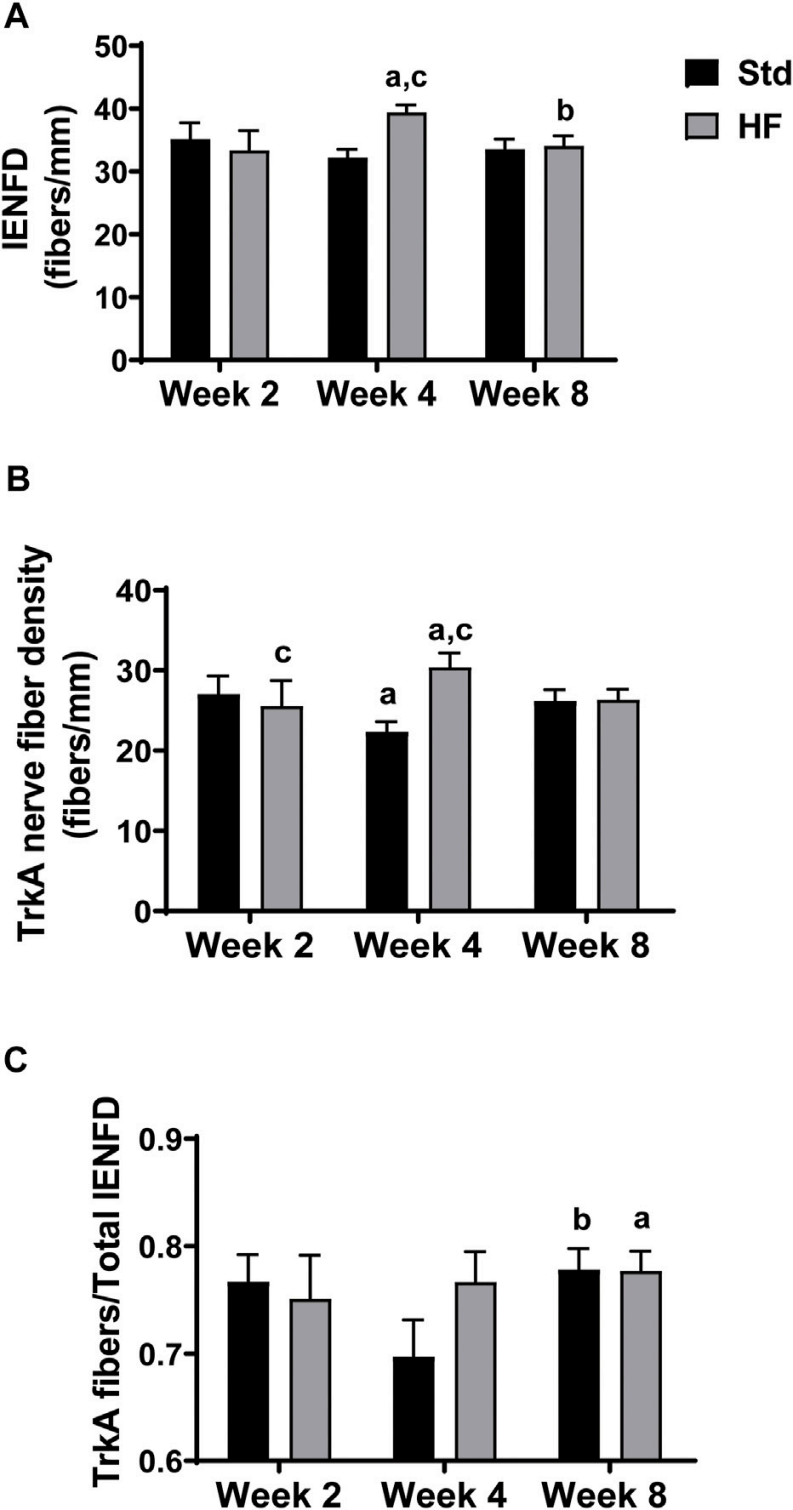
Epidermal Innervation. Intraepidermal nerve fiber density (IENFD) includes all PGP 9.5 positive fibers that cross the epidermal-dermal border **(A)**, TrkA nerve fiber density includes all TrkA positive fibers that cross the epidermal-dermal border **(B)**, and TrkA: IENFD ratio **(C)**. IENFD and TrkA nerve fibers peak and are greater than Std after 4 weeks of HF feeding. ^a^ *p* < 0.05 vs wk 2 within diet, ^b^ *p <* 0.05 vs wk 4 within diet, and ^c^ *p* < 0.05 for Std vs HF within the week. Data are presented as mean ± SEM (*n* = 8–12 mice per group).

**FIGURE 4:**
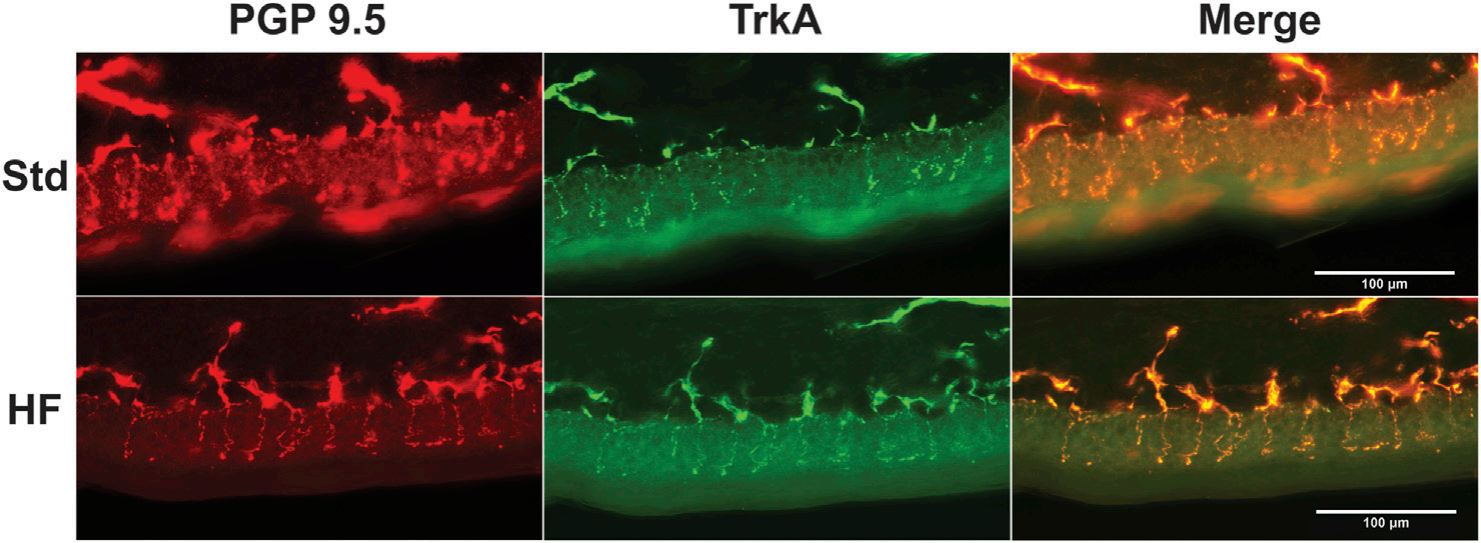
Representative Images of Epidermal Innervation. Fluorescent images show nerve fibers for Std (top panel) and HF (lower panel) after 8 weeks of dieteray intervention. Nerve fibers that crossed the epidermal/dermal border were quantified and expressed as fibers per mm. Anti-PGP 9.5 (red) labeled all nerve fibers and anti-TrkA (green) labeled TrkA nerve fibers.

After 4 weeks of dietary intervention, IENFD (Std: 32.2 ± 1.3, HF: 39.4 ± 1.2 fibers/mm, *p* = 0.004) and TrkA nerve fiber density (Std: 22.4 ± 1.3, HF: 30.4 ± 1.8 fibers/mm, *p* = 0.001 were greater in HF compared to Std (Figures 3A,B), but there was no difference between diet groups in IENFD (Std: 33.6 ± 1.5, HF: 34.1 ± 1.6 fibers/mm, *p* = 0.839) or TrkA nerve fiber density (Std: 26.2 ± 1.4, HF: 26.4 ± 1.3 fibers/mm, *p* = 0.939) after 8 weeks. Notably, IENFD and TrkA nerve fiber density increased in HF from week 2 (IENFD: 33.4 ± 3.1, TrkA: 25.6 ± 3.2 fibers/mm) to week 4 (IENFD: 39.4 ± 1.2, *p* = 0.004, TrkA: 30.4 ± 1.8 fibers/mm, *p* = 0.001) (Figures 3A,B). IENFD then decreased from week 4 (39.4 ± 1.2 fibers/mm) to week 8 (34.1 ± 1.6 fibers/mm, *p* = 0.028) in HF (Figure 3A). The TrkA: IENFD ratio was greater in Std week 8 (0.78 ± 0.02) compared to week 4 (0.70 ± 0.03, *p* = 0.039) and greater in HF at week 8 (0.78 ± 0.02) compared to week 2 (0.75 ± 0.04, *p* = 0.039) (Figure 3C). However, there were no significant differences between Std and HF in the TrkA: IENFD ratio (Figure 3C) at any time point. A representative image for one region of one tissue section from one mouse per each diet group after the 8 week intervention is shown in Figure 4.

### Inflammatory mediators

**FIGURE 5:**
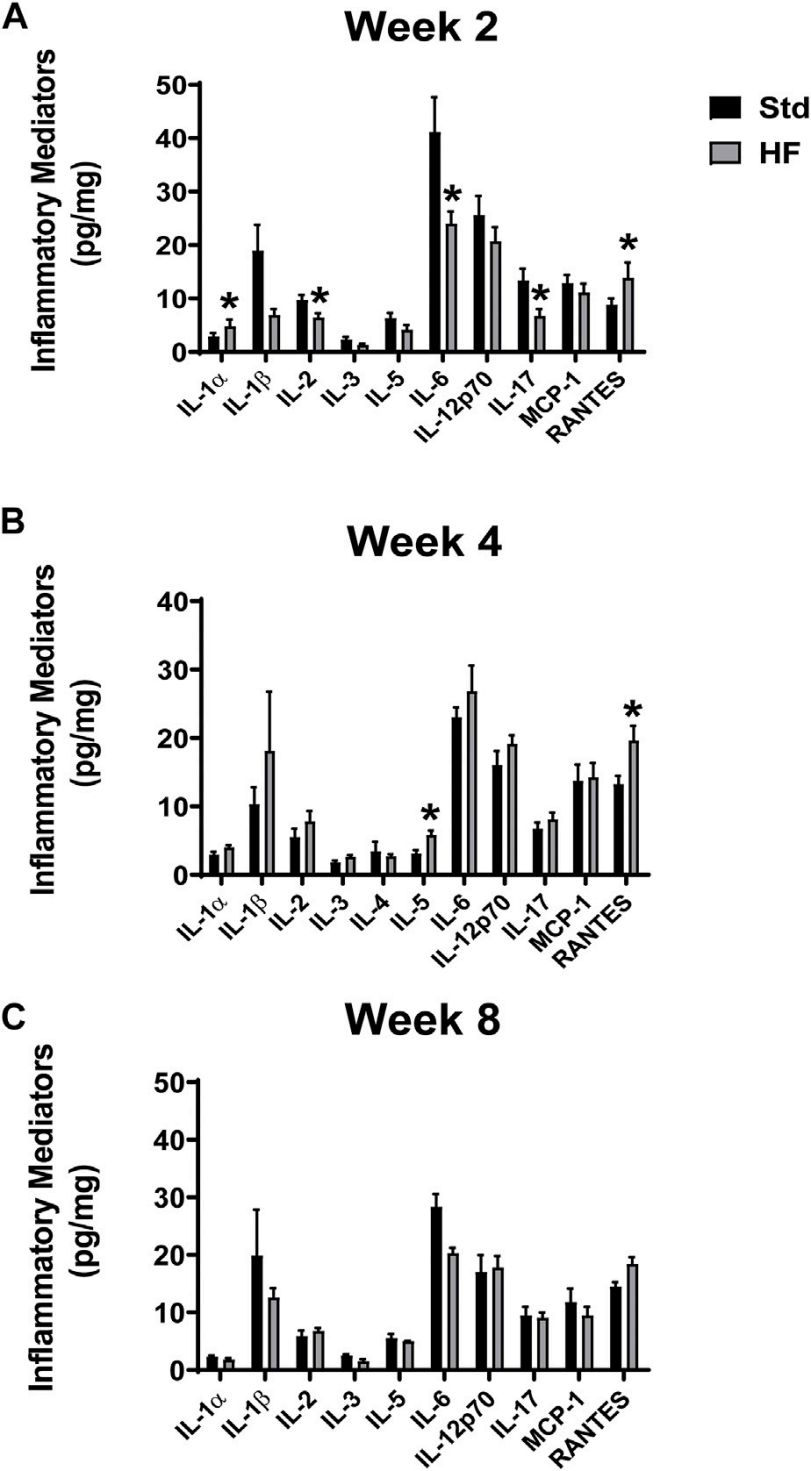
Inflammatory Mediators in the Lumbar DRG. Std vs HF after 2 weeks **(A)**, 4 weeks **(B)**, and 8 weeks **(C)** of dietary intervention. Data are presented as mean ± SEM (*n* = 3–12 mice per group). ^*^ *p* < 0.05 vs for Std vs HF. IL-10, IFN-γ, TNF-α, MIP-1a, GMCSF were below the limits of detection in the majority of the samples, thus means could not be calculated.

**FIGURE 6:**
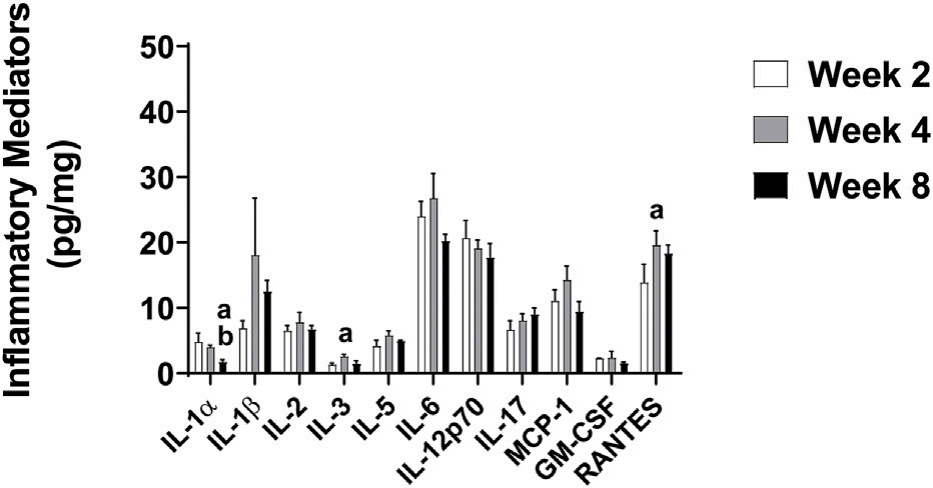
Inflammatory Mediators in the Lumbar DRG in HF Group after 2, 4, and 8 Weeks. Data are presented as mean ± SEM (*n* = 3–11 mice per group). ^a^ *p* < 0.05 vs wk 2, ^b^ *p <* 0.05 vs wk 4. IL-10, IFN-γ, TNF-α, MIP-1α were below the limit of detection in the majority of the samples, thus means could not be calculated.

IL-1α and RANTES were higher after 2 weeks of HF diet (IL-1α: 4.8 ± 1.3, RANTES: 13.9 ± 2.8 pg/mg) compared to Std (IL-1α: 2.9 ± 0.6, *p* = 0.031, RANTES: 8.8 ± 1.2 pg/mg, *p* = 0.04 (Figure 5A). In addition, IL-5 and RANTES were elevated after 4 weeks of HF diet (IL-5: 5.8 ± 0.7, *p* = 0.028, RANTES: 19.6 ± 2.2 pg/mg) compared to Std (IL-5: 3.1 ± 0.5, RANTES: 13.2 ± 1.2 pg/mg, *p* = 0.014) (Figure 5B). However, there were no significant differences between Std and HF after 8 weeks of the dietary intervention (Figure 5C). IL-3 and RANTES increased significantly in the HF group from 2 weeks (IL-3: 1.3 ± 0.3 pg/mg, RANTES: 13.9 ± 2.8 pg/mg) to 4 weeks (IL-3: 2.6 ± 0.3 pg/mg, *p* = 0.032, RANTES: 19.6 ± 2.2 pg/mg, 0.023) (Figure 6). In addition, IL-1α was significantly lower in HF at week 8 (1.8 ± 0.3 pg/mg) compared to week 2 (4.8 ± 1.3 pg/mg, *p* = 0.001) and week 4 (4.0 ± 0.3 pg/mg, *p* = 0.001) (Figure 6). Moreover, the majority of inflammatory mediators in HF peaked (although only a few reached statistical significance) at the 4-weeks timepoint and then decreased by 8 weeks (Figure 6).

## Discussion

Previous studies indicate nondiabetic rodents fed a high fat diet develop obesity (Obrosova et al., 2007; Watcho et al., 2010; Jaroslawska et al., 2021), dyslipidemia (Vincent et al., 2009; Guilford et al., 2011; Ozay et al., 2014; Zhang et al., 2018; Jaroslawska et al., 2021), and signs of PN including morphological changes in peripheral nerves (Zhang et al., 2018), reduced sensory and motor nerve conduction velocities (Vincent et al., 2009; Xu et al., 2014), altered thermal sensitivity (Obrosova et al., 2007; Guilford et al., 2011; Xu et al., 2014; Zhang et al., 2018), and mechanical hypersensitivity (Guilford et al., 2011; Xie et al., 2013; Zhang et al., 2018). However, the distinct mechanisms underlying high fat diet-induced PN have yet to be elucidated.

Previous studies from our laboratory detected increased neuronal inflammation after just 4 weeks of high fat feeding in mice that show mechanical hypersensitivity. However, neuronal inflammation was not present after 8 weeks of high fat feeding (unpublished data). In the current study, we aimed to determine if neuronal inflammation preceded or occurred in conjunction with behavioral and nerve fiber changes in high fat fed mice that are nondiabetic but insulin resistant. The current investigation was the first to assess neuronal inflammation and signs of PN including mechanical sensitivity and nerve fiber density (including IENFD and TrkA) at multiple timepoints in high fat fed mice.

Mice in the current study exhibited increased body mass and fasting glucose and insulin levels after 8 weeks of high fat feeding. Pro-inflammatory mediators in the lumbar DRG were greater after 2 weeks (IL-1α and RANTES) and 4 weeks (IL-5 and RANTES) of high fat feeding compared to mice fed the standard diet, but there were no significant differences in inflammatory mediators after 8 weeks of the diet intervention. Following a similar pattern, IENFD and TrkA nerve fiber density were greater in HF compared to Std after 4 weeks of high fat feeding, but these differences resolved after 8 weeks. Notably, increased inflammatory mediators in the peripheral nervous system preceded and accompanied increased nociceptive TrkA nerve fiber density in HF, suggesting a potential relationship between increased pro-inflammatory mediators and increased nociceptive nerve fiber density.

### High fat fed mice exhibit signs of insulin resistance

As expected, high fat feeding increased body mass and glucose and insulin levels compared to Std, but glucose levels were not high enough to indicate diabetes (fasting glucose approximately 230 mg/dl in mice). Insulin levels were elevated in HF compared to Std as early as week 2, followed by elevated glucose at week 4 and greater body mass at week 8. The effects of the high fat diet on body mass and metabolic hormones are consistent with previous studies (Obrosova et al., 2007; Watcho et al., 2010; Xu et al., 2014) that have used the high fat diet-induced insulin resistant mouse model for studying early-stage PN progression.

### High fat diet induces changes in epidermal innervation

IENFD and TrkA nerve fiber density was significantly greater in HF compared to Std after 4 weeks of high fat feeding, but these differences disappeared after 8 weeks of high fat feeding. While previous investigations in STZ-induced type 1 diabetic rodent models exhibit reduced IENFD (Christianson et al., 2003a; Kennedy and Zochodne, 2005; Ozaki et al., 2018), high fat fed mice show no change in IENFD (Obrosova et al., 2007; Guilford et al., 2011; Xu et al., 2014) or increased TrkA nerve fiber density (Groover et al., 2013; Zhang et al., 2018). Notably, previous studies showing no change in IENFD were after longer term high fat feeding: 8 weeks (Guilford et al., 2011), 14 weeks (Xu et al., 2014) or 16 weeks (Obrosova et al., 2007). We suspect an early brief onset of neuronal inflammation is associated with changes in epidermal innervation that initiate the development of behavioral signs of PN. If this idea is correct, then diet interventions greater than 8 weeks may fail to detect neuronal inflammation and changes in epidermal innervation despite the presence of behavioral signs of PN. In the current study, significant differences between Std and HF in IENFD and TrkA nerve fiber density occurred after just 4 weeks of high fat feeding and were resolved by week 8. TrkA fibers are a subset of total nerve fibers (represented by IENFD), thus increased IENFD is likely due to the specific increase in TrkA pain sensing nerve fibers. Although, the current cohort of mice did not exhibit statistically significant increased mechanical sensitivity compared to Std at any time point (see limitations), we still suspect this early (after 4 weeks) increase in nerve fiber density may peak in conjunction with the onset of increased mechanical sensitivity in high fat fed and/or obese models of PN.

For example, Cheng et al. reported that *db/db* type 2 diabetic mice experience increased mechanical sensitivity between 6 and 12 weeks of age and suggest that increased IENFD and TrkA nerve fiber density that occurs at this time is in part responsible for the development of mechanical hypersensitivity (Cheng et al., 2009; Cheng et al., 2012). Further, Groover et al. demonstrated that nondiabetic insulin resistant mice fed a high fat diet developed mechanical hypersensitivity, increased nerve growth factor (Paul et al., 2016) in the DRG and hindpaw footpad, and TrkA fiber density compared to Std. TrkA is the high affinity receptor for nerve growth factor and fibers that express TrkA are small, peptidergic fibers that express nociceptive peptides (Kashiba et al., 1996). Nerve growth factor has been shown to elicit hyperalgesia (Lewin et al., 1993) and modulate inflammatory pain (Woolf et al., 1997; Saade et al., 2000), and has been suggested to play a critical role in the development of diabetic PN. Taken together, prior studies illustrate a relationship between increased inflammatory mediators and increased cutaneous TrkA innervation associated with increased nociception characterized by mechanical hypersensitivity. Results of the current study are consistent with this idea and demonstrate a link between increased inflammatory mediators and increased TrkA, but failed to detect mechanical sensitivity in high fat fed mice.

### Peripheral nervous system inflammation in high fat fed mice

Despite extensive evidence that chronic hyperglycemia is the primary insult that leads to the pathogenesis of diabetic PN (Feldman et al., 1997); even when blood glucose levels are tightly controlled, neuropathic symptoms still persist (Azad et al., 1999). In support of this idea, results from the Eurodiab Trial indicate hypertension, serum lipids, and body mass index were each independently associated with increased risk of developing PN in type 1 diabetic patients who did not have PN at the beginning of this large longitudinal population based study (Leiter, 2005). Taken together, this evidence indicates that factors independent of hyperglycemia (i.e. dyslipidemia, inflammation, obesity, etc.) may contribute to the development of PN, in prediabetic and diabetic patients.

Inflammation has been proposed as a key mechanism contributing to PN progression (Zeng et al., 2018; Zhang et al., 2018) and several studies have reported elevated inflammatory factors in the serum of patients with diabetic PN (Matsuda et al., 2004; Zhu et al., 2015; Zeng et al., 2018) and animal models of PN (Yamakawa et al., 2011; Ozay et al., 2014). Previous investigations have specifically identified a relationship between TNF-α and PN (Matsuda et al., 2004; Yamakawa et al., 2011; Hussain et al., 2013; Ozay et al., 2014; Zhu et al., 2015). Matsuda et al. reported age-corrected sensory nerve conduction velocity was negatively correlated with TNF-α in type 2 diabetic patients (Matsuda et al., 2004). Yamakawa et al. demonstrated that inactivation of TNF-α ameliorated motor and sensory nerve conduction deficits and thermal hypoalgesia in diabetic mice (Yamakawa et al., 2011). In addition, nondiabetic mice fed a high fat diet show a significant increase in TGF-β and TNF- α accompanied by reduced myelin thickness and nerve fiber diameter (Ozay et al., 2014). Further, evidence in humans indicates the proinflammatory phase during prediabetes may be an initiating factor that contributes to the development of PN (Zeng et al., 2018). However, only one previous study has assessed inflammation within the peripheral nervous system (Zhang et al., 2018) as we have done in this study.

In the current study, pro-inflammatory mediators in the lumbar DRG were greater in HF after 2 weeks (IL-1α and RANTES) and 4 weeks (IL-5 and RANTES) compared to mice fed the standard diet. RANTES and IL-3 significantly increased from 2 to 4 weeks in the HF group. After 8 weeks of high fat feeding, IL-1α was significantly lower compared to weeks 2 and 4 in the HF. Similar to the pattern seen in Il-1α, many of the inflammatory mediators appeared to peak at week 4 and were lower by week 8 in HF, although few reached statistical significance. In addition, there were no significant differences between Std and HF after 8 weeks. Our findings are consistent with results in genetically obese, *ob/ob* mice that showed increased inflammatory gene expression in genes at 5 weeks compared to 8 weeks of age (O'Brien et al., 2015). In addition, Xu et al. reported increased expression of 6 proinflammatory genes in the white adipose tissue after 3 weeks of high fat feeding (Xu et al., 2003). Taken together, these results suggest an early inflammatory response subsequently followed by anti-inflammatory process that restores inflammatory mediators to near baseline levels. Interestingly, the presence of inflammation after 2 and 4 weeks of a high fat diet which preceded and accompanied the increase in IENFD and TrkA nerve fiber density suggests the initial inflammatory response may initiate changes in epidermal innervation which could lead to increased nociception.

Notably, the proinflammatory chemokine RANTES was greater after both 2 and 4 weeks of high fat feeding compared to standard fed mice. Previous studies indicate increased RANTES in adipose tissue of obese high fat fed mice compared to lean controls (Wu et al., 2007) and non-diabetic obese humans exhibit increased gene expression of RANTES and its receptor, CC-chemokine receptor type 5 (CCR5) in subcutaneous adipose tissue compared to lean humans (Wu et al., 2007). RANTES is chemotactic for monocytes and eosinophils, is produced by T lymphocytes and macrophages, causes the release of histamine from basophils, and activates eosinophils (Zeng et al., 2022). Doupis et al. demonstrated elevated RANTES in the serum of diabetic patients with PN compared to diabetic patients without PN (Doupis et al., 2009), suggesting a potential role for RANTES in PN.

In addition, an *in vitro* study in rat cerebral cortical tissue exposed to menadione resulted in increased cell death with a corresponding increase in both IL-1α and RANTES compared to untreated control cells (Tripathy and Grammas, 2009), supporting a role for IL-1α and RANTES in the inflammatory response in neural tissue. Furthermore, RANTES has been heavily implicated in the development of painful PN. In a partial sciatic nerve litigation model, upregulation of the RANTES receptor (CCR5) was observed with a corresponding increase in mechanical sensitivity while mechanical sensitivity was attenuated when the CCR5 antagonist, D-ala-peptide T-amide, was administered (Saika et al., 2012). RANTES is an important signaling molecule for recruitment of activated macrophages and T cells (Wu et al., 2007). Following partial sciatic nerve ligation injury, C-C motif chemokine ligand 5 (CCL5, aka RANTES) knockout mice had significantly less macrophage and pro-inflammatory cytokine infiltration in damaged nerves and less behavioral hypersensitivity than wild-type controls, suggesting that RANTES plays an integral role in regulating painful behavior and the inflammatory response in the peripheral nervous system (Liou et al., 2012).

Interestingly, eosinophils are an abundant source of RANTES and nerve growth factor, both of which have been shown to be interact with peripheral nerves. Eosinophils are prominent sources of inflammatory cytokines and chemokines, comprise 2–10% of peripheral leukocytes, and are involved in neuroimmune interactions that regulate the functional activity of peripheral nerves. (Raap and Wardlaw, 2008). Once activated, eosinophils release several cytokines, including but not limited to IL-5 and IL-1α (Raap and Wardlaw, 2008). In the current study, RANTES, IENFD, and TrkA nerve fiber density all increased in the HF group from 2 to 4 weeks with a subsequent decrease by the 8-weeks timepoint. IL-1α and IL-5 were both greater in HF compared to Std at 2 and 4 weeks, respectively. Although eosinophils release IL-5, they are also responsive to IL-5 (from lymphocytes), which is required for maturation of eosinophils (Raap and Wardlaw, 2008) and RANTES is a key chemokine that is required for eosinophil migration and chemotaxis (Wardlaw, 1999). Although NGF was not measured in the present investigation, perhaps the elevation in IL-5 and RANTES in the lumbar DRG helped drive eosinophil release of NGF in the DRG which could modulate TrkA nerve fiber density in the epidermis. Increased TrkA nerve fiber density has been shown to be accompanied by increased NGF in the lumbar DRG in mice fed a HF diet for 12 weeks Thus, neuronal inflammation, via upregulation of NGF, may alter nociceptive input in the DRG by increasing TrkA nerve fiber density and lead to increased mechanical sensitivity in high fat diet-induced prediabetic models of PN.

### Limitations

Results of the current study should be interpreted with consideration of the limitations. In rodent models, PN is typically characterized by one or a combination of the following: mechanical sensitivity, nerve conduction velocity, IENFD, beam walk, and/or thermal sensitivity. The fact PN was characterized by just mechanical sensitivity and IENFD may be considered a limitation. However, previous studies in high fat fed mice have showed inconsistent effects on sensory and motor nerve conduction velocity, thermal sensitivity, and performance on beam walk, while high fat fed mice consistently demonstrate mechanical hypersensitivity (Obrosova et al., 2007; Watcho et al., 2010; Groover et al., 2013; Xie et al., 2013; Zhang et al., 2018). Although thermal sensitivity assessment has been suggested as a preferred method to characterize neuropathy in mouse models of diabetic PN, high fat diet induced models of PN are phenotypically different than diabetic models and exhibit inconsistent effects on thermal sensitivity (Xu et al., 2014). Some studies in high fat fed mice show no change in thermal sensitivity (Groover et al., 2013; Cooper et al., 2018) while others show hyperalgesia (Pittenger et al., 2005; Singleton et al., 2005; Xu et al., 2014) or hypoalgesia (Obrosova et al., 2007; Vincent et al., 2009). Von Frey behavioral testing to assess hindpaw sensitivity is a widely accepted method characterize PN in rodents and is analogous to aspects of quantitative sensory tests in human patients (Biessels et al., 2014). Because mechanical hypersensitivity stands out as a hallmark feature of high fat diet induced PN, it is the preferred method of PN assessment high fat fed nondiabetic mice. Thus these experiments were specifically designed to assess the timeline of changes in mechanical sensitivity in conjunction with changes in epidermal innervation and neuronal inflammation, thus additional assessments of PN were not necessary to test the hypothesis.

Perhaps the greatest limitation of the current study is the failure to demonstrate statistically significant mechanical hypersensitivity in this cohort of high fat fed mice. However, mechanical hypersensitivity in high fat fed mice has been consistently demonstrated in previous studies from our work in different testing facility and others in the field (Obrosova et al., 2007; Watcho et al., 2010; Groover et al., 2013; Xie et al., 2013; Zhang et al., 2018). We attribute the lack of significant mechanical hypersensitivity to the behavioral testing environment. Our current mouse behavioral testing facility is a small room within a large exercise physiology lab in which human metabolic testing occurs along with student work in large laboratory classes. Although the behavior room has a heavy door, dim lights, and white noise, it cannot match the effects of a soundproof chamber inside a rodent behavioral testing facility. We expect that mechanical sensitivity was slightly increased in all mice due to noise pollution, thus the withdrawal threshold of Std was inappropriately elevated thus preventing the detection of statistically significant differences between Std and HF. Prior studies showing increased mechanical sensitivity (using this same von Frey percent withdrawal method) in high fat fed mice were performed in a sound proof chamber inside a rodent behavioral testing facility (Guilford et al., 2011; Groover et al., 2013; Cooper et al., 2018). Because mouse behavioral testing is typically and ideally performed in a soundproof chamber specifically designed to eliminate rodent stress due to external noise, it is plausible that the majority of prior research studies that report significant mechanical hypersensitivity in high fat fed mice were more sensitive changes in sensitivity due to a more controlled testing environment.

In addition, it could be argued that the von Frey “up-down” method which utilizes a range of several filaments to assess mechanical sensitivity would be more effective at detecting statistically significant differences compared to the use of a single 1.4 g von Frey filament (withdrawal percentage method) as we have done in the current study. However, in previous studies of high fat diet induced PN, we performed both the “up down” method and withdrawal percentage method on separate days and found the withdrawal percentage method to be more sensitive at detecting differences in mice fed a standard vs high fat diet. Although the data from the up-down method is unpublished, this beta testing informed the decision to use the withdrawal percentage method in this high fat fed model of PN (Guilford et al., 2011). Furthermore, the withdrawal percentage method is a widely accepted method for characterizing PN in mouse models of PN (Wright et al., 2007; Guilford et al., 2011; Jack et al., 2011) and has been shown to produce similar results to the up-down method in the same cohort mouse models of PN (Christianson et al., 2003b).

Lastly, assessment of neuronal inflammation in the lumbar DRG is limited by the minimal amount of protein that can be extracted from this tissue. Although multiplex ELISA is the method that allows the most comprehensive assessment of inflammatory mediators in this tissue, five analytes fell under the limits of detection and had to be omitted from the results. The quantification of inflammatory gene expression in the lumbar DRG may have added to this study, but an additional cohort of mice would be required for this analysis. To our knowledge, only one previous study has assessed neuronal inflammation in a high fat fed rodent model of PN, and multiplex ELISA was the chosen method.

## Conclusion

The results of the current study suggest that neuronal inflammation is associated with increased cutaneous nociceptive nerve fiber density in a high fat fed insulin resistant mice. Despite the lack of significant mechanical allodynia in the current study, it is plausible that increased nociceptive nerve fiber density is driven by early inflammation and leads to behavioral signs of neuropathy in other high fat fed mouse models of PN. A diet excess in calories or fat that is accompanied by obesity and glucose intolerance may initiate an inflammatory response that subsequently impacts the peripheral nervous system in humans. These findings highlight the notion that inflammation occurs in the early stages of obesity and glucose dysregulation, and inflammation may be a key mechanism responsible for changes in epidermal innervation that subsequently leads to the development of PN in insulin resistant humans. The clinical significance of these results lies in the possibility that therapies targeted at preventing or reducing neuronal inflammation in early stages of obesity and insulin resistance may hold therapeutic potential for the prevention or treatment of PN.

## Data Availability

The original contributions presented in the study are included in the article/Supplementary Materials, further inquiries can be directed to the corresponding author.
